# An Anthropomorphic Digital Reference Object (DRO) for Simulation and Analysis of Breast DCE MRI Techniques

**DOI:** 10.3390/tomography8020081

**Published:** 2022-04-02

**Authors:** Leah Henze Bancroft, James Holmes, Ryan Bosca-Harasim, Jacob Johnson, Pingni Wang, Frank Korosec, Walter Block, Roberta Strigel

**Affiliations:** 1 Department of Radiology, University of Wisconsin-Madison, 600 Highland Ave, Madison, WI 53792, USA; jim-holmes@uiowa.edu (J.H.); jmjohnson33@wisc.edu (J.J.); fkorosec@wisc.edu (F.K.); wfblock@wisc.edu (W.B.); RStrigel@uwhealth.org (R.S.); 2 Department of Radiology, University of Iowa, 169 Newton Road, Iowa City, IA 52333, USA; 3 Holden Comprehensive Cancer Center, University of Iowa, 169 Newton Road, Iowa City, IA 52333, USA; 4 Department of Imaging Physics, Sanford Health, 801 Broadway North, Fargo, ND 58102, USA; Ryan.Bosca-Harasim@SanfordHealth.org; 5 Department of Medical Physics, University of Wisconsin-Madison, 1111 Highland Avenue, Madison, WI 53705, USA; b9803216@gmail.com; 6 Department of Biomedical Engineering, University of Wisconsin, 1415 Engineering Drive, Madison, WI 53706, USA; 7 Carbone Cancer Center, University of Wisconsin, 600 Highland Avenue, Madison, WI 53792, USA

**Keywords:** digital reference object, quantitative imaging biomarker, DCE MRI, breast MRI, numerical simulation

## Abstract

Advances in accelerated magnetic resonance imaging (MRI) continue to push the bounds on achievable spatial and temporal resolution while maintaining a clinically acceptable image quality. Validation tools, including numerical simulations, are needed to characterize the repeatability and reproducibility of such methods for use in quantitative imaging applications. We describe the development of a simulation framework for analyzing and optimizing accelerated MRI acquisition and reconstruction techniques used in dynamic contrast enhanced (DCE) breast imaging. The simulation framework, in the form of a digital reference object (DRO), consists of four modules that control different aspects of the simulation, including the appearance and physiological behavior of the breast tissue as well as the MRI acquisition settings, to produce simulated k-space data for a DCE breast exam. The DRO design and functionality are described along with simulation examples provided to show potential applications of the DRO. The included simulation results demonstrate the ability of the DRO to simulate a variety of effects including the creation of simulated lesions, tissue enhancement modeled by the generalized kinetic model, T1-relaxation, fat signal precession and saturation, acquisition SNR, and changes in temporal resolution.

## 1. Introduction

Quantitative imaging continues to play an increasingly important role in informing the diagnosis and treatment of diseases including cancer. Recent advances in magnetic resonance imaging (MRI) data acquisition and reconstruction techniques provide an opportunity to overcome the traditional tradeoffs between scan time, resolution, field-of-view, and image quality by using highly undersampled acquisition trajectories paired with sophisticated reconstruction approaches. Validating and standardizing these approaches for use in quantitative imaging is critical for a more widespread clinical uptake of these methods. The adoption of these methods remains slow in part due to the difficulty in validation and standardization. Addressing gaps in validation and standardization has become a focus of several recent initiatives including the Radiological Society of North America’s (RSNA) Quantitative Imaging Biomarkers Alliance (QIBA), the National Cancer Institute’s Quantitative Imaging Network, and the International Society for Magnetic Resonance in Medicine’s (ISMRM) study group on Reproducible Research and the Open Science Initiative for Perfusion Imaging (OSIPI) [1,2,3].

Breast cancer remains the most commonly diagnosed cancer, excluding skin cancer, in American women and is the second leading cause of cancer death [4]. Quantitative imaging biomarkers (QIB) derived from dynamic contrast enhanced (DCE) MRI, made possible through the use of accelerated acquisition and reconstruction techniques, have shown promise in applications such as breast cancer detection [5], treatment planning [6] and the evaluation of response to therapy [7]. When utilizing sophisticated pharmacokinetic models [8,9,10] to produce QIBs from DCE MRI images, the DCE acquisition must achieve a temporal resolution of 20 s or less in order to minimize bias and variance in the measured pharmacokinetic parameters [11]. Moreover, spatial resolution capable of representing tumor morphology and heterogeneity, with typical in-plane resolution at or below 1 × 1 mm^2^, is critical for breast cancer imaging applications [12,13,14]. Meeting these competing demands requires the use and rigorous validation of accelerated MRI data acquisition and reconstruction strategies.

Accelerated MRI data acquisition and reconstruction techniques that have been applied to DCE MRI include parallel imaging [15], undersampling with view sharing [5,16,17,18,19,20,21,22], low-rank matrix recovery approaches [23,24] and compressed sensing reconstructions [25,26,27,28,29,30,31]. These techniques achieve acceleration through sampling data below the Nyquist frequency. Images of acceptable quality are recovered from undersampled data by imposing assumptions on the data during the reconstruction process. An accurate reconstruction of the underlying anatomy and physiology depends upon these assumptions not being violated. For example, view-sharing techniques have been shown to suffer from temporal blurring due to data sharing between time points [30] and low rank approaches can fail when faced with rapidly changing tissue kinetics or high degrees of undersampling. Establishing the robustness and limitations of accelerated acquisition and reconstruction techniques for a given imaging application remains difficult. In vivo studies are often limited in numbers and the range of different physiological conditions they can represent. Furthermore, the underlying ground truth is often unknown, making rigorous validation difficult. This is further exacerbated in the setting of breast imaging where multiple contrast injections cannot be performed in a day due to the contrast agent wash-out period, thus preventing back-to-back comparisons of acquisition techniques.

Sufficiently realistic numerical phantoms or digital reference objects (DRO) offer a known ground truth to which advanced reconstruction methods can be compared. Highly detailed numerical phantoms have been developed for MRI image analysis applications in the brain [32,33,34,35,36,37,38,39,40], liver [41,42], prostate [41], and for cardiac applications [43,44,45]. Specific DROs for breast imaging analysis have also been developed including those for mammography [46,47,48,49], computed tomography (CT) [50], breast tomosynthesis [47], and breast microwave imaging [51,52]. To date, there has been less work on the development of a numerical phantom for breast MRI applications. Le et al. described a digital phantom as part of their work in accelerated DCE breast imaging [53]. However, no DRO has been described that provides a more realistic simulation of the spatially and temporally complex imaging environment encountered in DCE breast imaging. Such features can be critically important in assessing the performance of more sophisticated acquisition and reconstruction methods that exploit spatial-temporal correlations such as compressed sensing. 

In this work, we describe the development of a simulation framework in the form of a breast DRO for use in analyzing and optimizing DCE breast MRI acquisition and reconstruction techniques. The phantom includes characteristics important to DCE breast MRI, such as simulated enhancing lesions of different sizes and shapes surrounded by normal fibroglandular breast tissue with varying levels of background parenchymal enhancement (BPE) [54] and non-enhancing fat tissue. Prior studies have demonstrated the utility of the phantom in the validation of novel acquisition and reconstruction methods [31,55,56,57,58,59,60,61]; however, this is the first publication to provide a detailed description of the development, architecture, and functionality of the phantom. The objective of this numerical phantom is to provide a tool for simulating a realistic DCE breast MRI imaging environment that allows simulation results to be compared to a known “truth” in a highly controlled setting. 

## 2. Materials and Methods

### 2.1. Breast MRI DRO Overview

A DRO was constructed using a modular design to readily allow users to test the following imaging scenarios:

1. Different MRI input images including anatomy and chemical species content
2. Different coil sensitivity profiles
3. Contrast enhancement based on user desired kinetic models
4. Simulation of MRI physics
5. Simulation of k-space sampling in both time and space.

**Figure 1:**
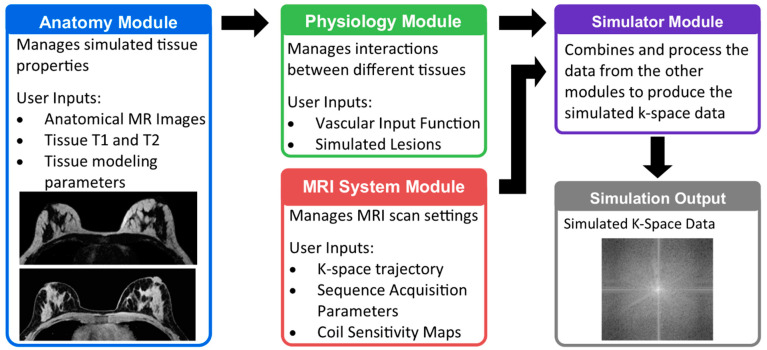
Overview of the structure of the digital reference object (DRO) for simulation of breast dynamic contrast enhanced (DCE) MRI. The DRO is split into four modules, each of which manages different aspects of the simulation. The Anatomy Module contains functions for setting up and managing the appearance and behavior of the tissues to be simulated. A simulation may contain more than one class of tissue, for example water- and fat-based tissues. Interactions between tissues, as well as responses common to all tissues such as the vascular input function, are managed by the Physiology Module. Parameters related to the MRI scan settings to be simulated are managed by the MRI system Module. These inputs are provided to the Simulator Module that then generates the final simulated k-space data.

The DRO was designed to consist of four modules that provide control over specific aspects of the simulation platform. These include (1) the Anatomy Module, which contains specific tissue distributions and properties, (2) the Physiology Module, which controls interactions within the Anatomy Module, (3) the MRI System Module, which contains the MRI scan settings to be simulated, and (4) the Simulator Module, which produces the simulated k-space data. An overview of the DRO modules as well as their inputs and outputs are shown in Figure 1.

Each of these modules is currently implemented using MATLAB [62] and are available for download at https://github.com/lchenze/DRO_Breast_DCE_MRI. The modules are described in greater detail below. Example simulations were performed to demonstrate the functionality of each module. 

### 2.2. Anatomy Module

An anthropomorphic representation of the breast is obtained using in vivo image data collected from volunteers undergoing breast MRI. As the breast contains a heterogenous distribution of fat and fibroglandular tissue, the DRO is set up to operate on separated fat and water images. Different chemical species, such as fat and water, are treated as different tissue classes by the Anatomy Module. Each tissue class is assigned a unique spectral model that defines the precession of the MRI signal. The fat and water separated anatomical images define the spatial extent of each chemical species. Tissue classes may be further divided into tissue subtypes with each subtype having a unique T_1_ and T_2_ value, as well as relevant physiological or model specific parameters to be used by the Simulator Module described below. Examples of potential water tissue subtypes include breast fibroglandular tissue and the muscle of the chest wall. The spatial extent of each tissue subtype is defined by a three-dimensional mask that is the same size as the parent tissue. 

Background parenchymal enhancement [54,63] (BPE) is frequently observed after contrast administration during in clinical breast exams and has the potential to obscure lesions of interest. To simulate this physiological effect, the Anatomy Module contains a method to divide the fibroglandular tissue subtype into regions that can be assigned unique physiological or model specific parameters to produce the desired level of background parenchymal enhancement in the Simulator Module. In actual breast exams, the amount of BPE is highly variable from patient to patient [64]. The DRO, therefore, allows the fraction of the fibroglandular tissue assigned to the BPE region to vary from 0 to 100%. The selection of voxels assigned to the BPE region is weighted such that fibroglandular tissue in the upper outer quadrants of the breast are more likely to be included in the BPE regions, reflecting what is seen in vivo [65]. 

### 2.3. Physiology Module

The Physiology Module controls the interactions between different tissues and contains parameters related to physiology, such as the vascular input function which describes the expected concentration of the gadolinium contrast agent in the vascular space over time following injection. The generation and insertion of simulated lesions into the breast DRO is controlled by the Physiology Module as lesions can be inserted into any location in the breast. 

The Physiology Module contains methods to generate four generalized lesion shapes (round, lobulated, irregular and spiculated) representing the four different classes of mass morphologies defined in the Breast Imaging Reporting and Data System (BIRADS) lexicon [54]. The round, lobulated and irregular lesion morphologies are generated using a series of randomly placed overlapping spheres. The spicules used in the spiculated lesion morphology are generated using the methodology presented in de Sisternes et al. [48]. User input is required to define certain lesion characteristics such as size and location, while other features, such as the number and length of spicules, may be user controlled or assigned pseudo-randomly at the time of lesion generation. The spatial extent of the lesion tissue subtype is defined by a three-dimensional mask, similar as to what is done for other tissue subtypes, but the voxels contained in the mask are assigned new grayscale values matching the mean and standard deviation of the fibroglandular tissue in the base anatomical images. This is done to allow lesions to be placed in regions of the breast containing fat tissue. 

Lesions may be made up of several tissue subtypes with each tissue subtype represented by a unique set of modeling parameters. A homogeneously enhancing lesion is represented by only one tissue subtype, while a heterogeneously enhancing lesion is represented by two or more. The spatial extent of a heterogeneously enhancing lesion tissue subtype may be defined manually by the user or a pseudo-random assortment of voxels within the lesion can be assigned to the tissue subtype by the Physiology Module. Rim-enhancement is a clinically meaningful form of heterogeneous enhancement associated with malignancy. To simulate rim-enhancement, the outer edges of a lesion are assigned to a separate tissue subtype by the Physiology Model.

### 2.4. MRI System Module

The MRI system Module contains the parameters required to generate the desired MRI signal. The MRI signal magnitude (*S_i_*) is determined using the steady state spoiled gradient echo (SPGR) signal model, ignoring *T*_2_* effects:

(1) $$S_{i}=S_{0}\frac{\sin\alpha \left(1-e^{-TR/T_{1}}\right)}{1-e^{-TR/T_{1}}\cos\alpha }$$

where *S*_0_ is the equilibrium magnetization signal intensity, α is the flip angle, *TR* is the repetition time, and *T*_1_ is the longitudinal relaxation time of the tissue. *S*_0_ is assumed to be the signal intensity defined in the Anatomy Module. User input is required to define the parameters for flip angle and TR as well as other relevant MRI parameters including field strength, bandwidth, k-space sampling pattern, desired fat suppression method if implemented at the time of data collection, and contrast agent relaxivity, if applicable. Coil sensitivity maps may be included to allow for the simulation of parallel imaging. 

The user supplied k-space sampling pattern can either be Cartesian or non-Cartesian and must be paired with a timing vector defining the start time of each TR. At the time of simulation, Cartesian data points are obtained using a standard fast Fourier transform (FFT). In the case of Non-Cartesian data, the DRO will use the 3D non-uniform Fourier Transform described by Greengard et al. [66] and implemented by Ferrara [67]. The user may also designate the use of an alternate transform function such as the 3D non-uniform Fourier Transform implemented by Fessler [68]. Additional scan parameters such as the echo time (TE), the number of echoes, the maximum k-space frequency and the spacing of k-space data points are controlled implicitly by the k-space sampling pattern supplied by the user.

The MRI System Module allows for simulation of a chemically selective fat saturation pulse as described in Foo et al. [69]. For the purpose of the simulation, the system is assumed to be in the steady state and the flip angle of the data acquisition pulse is assumed to be small. The equilibrium longitudinal magnetization of the fat signal prior to each inversion pulse $(M_{zEq})\;$is then calculated as:

(2) $$M_{zEq}=\frac{\left(1-e^{\left(-\frac{TR_{IR}}{T_{1}}\right)}\right)}{1-e^{-\frac{TR_{IR}}{T_{1}}}\cos\alpha_{IR}}$$

where $TR_{IR}$ is the repetition time of the periodic inversion (*IR*) pulse, $T_{1}$ is the $T_{1}$ value assigned to the fat tissue, and $\alpha_{IR}$ is the flip angle of the *IR* pulse. The amount of longitudinal magnetization for the fat tissue available prior to each imaging excitation pulse ($M_{z}\left(t\right))$ is then calculated as:

(3) $$M_{z}\left(t\right)=1-M_{zEq}\cos\alpha_{IR}e^{-t_{pulse}/T_{1}}$$

where $t_{pulse}$ is the time since the last inversion pulse. $M_{z}\left(t\right)$ is then applied as a scaling factor to $S_{i}$ given in Equation (1) to calculate the final fat signal magnitude following the imaging excitation pulse.

### 2.5. Simulator Module

**Figure 2:**
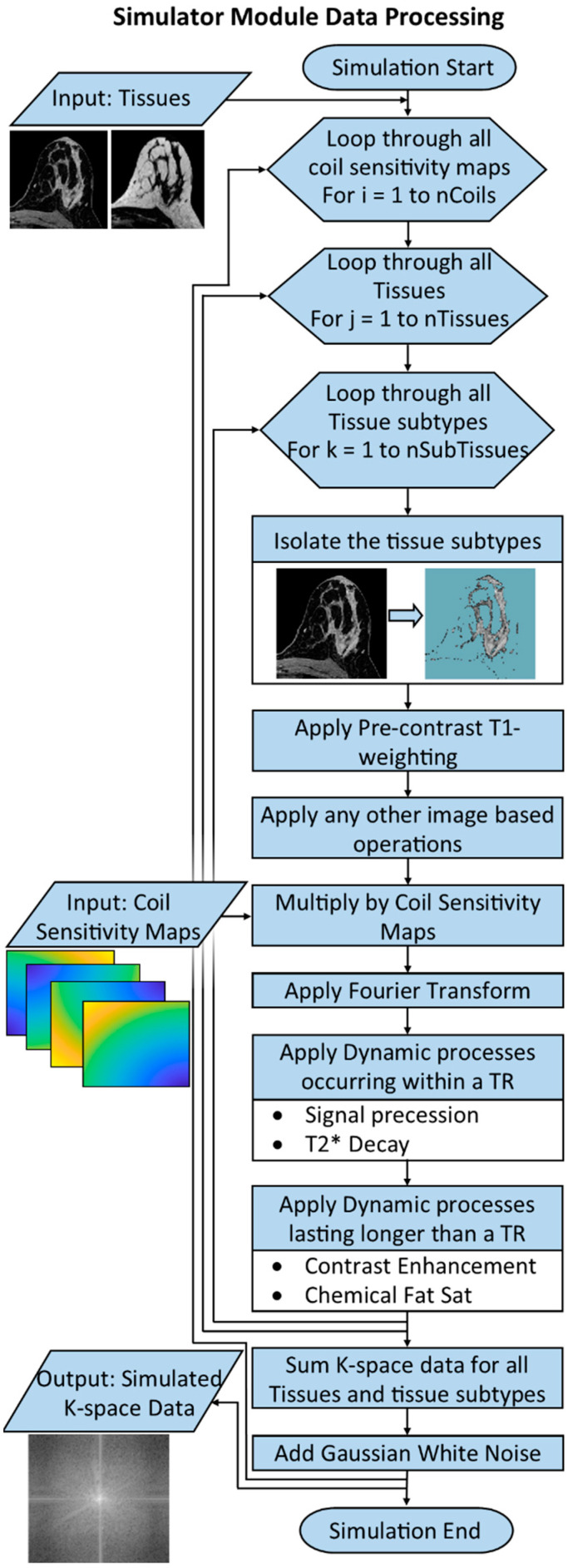
Flow chart illustrating how the Simulator Module processes the input data to produce the simulated k-space output. Tissues contained in the Anatomy Module, such as fat and water, are broken down into their tissue sub-types and processed separately. Static image space based processing, including applying T_1_ weighting consistent with the selected MR System parameters, is applied first. The Fourier transform is applied; then dynamic processing is applied in Fourier space. Dynamic processing may either operate on the data over the course of a single TR such as signal precession, or may operate over several TRs such as the simulated contrast uptake curves. Finally, the k-space data from all tissues and tissue sub-types are recombined and returned to the user for reconstruction.

The Simulator Module produces simulated k-space data for a DCE breast MRI scan based upon the data contained in the Anatomy, Physiology and MRI System Modules. An overview of the processing performed by the Simulator Module is shown in Figure 2. The Simulator Module takes the base anatomical images, including any simulated lesions, from the Anatomy Module. Image space processing is performed first to produce the desired image contrast of the MRI images at time t = 0 for the simulation. This includes operations such as applying appropriate pre-contrast MRI signal intensity based on the parameters contained in the MRI System Module (field strength, flip angle and TR) and the Anatomy Module (T_1_ relaxation time), and applying weighting required to simulate individual coil elements using sensitivity maps provided by the MRI System Module. If coil sensitivity maps are included, the remainder of the processing is performed separately for each coil element. Next, dynamic k-space processing is applied separately for each tissue class and each tissue subtype. The appropriate voxels belonging to a single tissue subtype are isolated and transformed into k-space.

K-space processing is performed in a series of steps. First, the desired k-space data points are generated using the appropriate Fourier transform. Next, dynamic changes occurring within a single TR are applied by multiplying the k-space data by a weighting function with real and imaginary components. Such processes would include signal precession due to different spectral models (i.e., precession of the fat signal) or signal decay due to T2* effects. Finally, the k-space data are multiplied by a weighting function corresponding to the contrast enhancement curve model specified by the user and the modeling parameters assigned to each tissue type. K-space data from each sub-tissue and tissue class are then combined to produce the final k-space data array. White Gaussian noise is added to the data, in a channel-by-channel fashion for multi-coil data, and the data are returned to the user as an array containing data for each k-space point specified in the sampling pattern. In the case of multiple coil elements, an array of data is returned for each coil element. 

### 2.6. Example Simulations Using the Breast DRO

#### 2.6.1. Simulation 1

The Anatomy Module described above was used to perform a simulation with the settings given below. Base anatomical images for the DRO were generated from volunteers imaged with a T_1_-weighted, chemical shift encoded (CSE) sequence. The DRO is relatively insensitive to the exact acquisition parameters used to generate the base anatomical images as long as the output images include a reasonable representation of the fat and water tissues present. The base anatomical images utilized in this work were acquired as follows: volunteers were imaged on a 1.5T scanner (Optima 450w, GE Healthcare, Waukesha, WI, USA) using an 8-channel breast array (GE Healthcare). This study was approved by the Institutional Review Board (IRB) at our institution and complied with the Health Insurance Portability and Accountability Act (HIPAA). T_1_-weighted, CSE images were obtained and processed using the vendor-supplied versions of two-echo CSE-MRI [70] (VIBRANT Flex, GE Healthcare) or three-echo CSE-MRI [71] (IDEAL, GE Healthcare). Settings similar to those used in standard clinical breast exams at our institution were used including a 32 × 32 cm^2^ axial field of view, 0.83 mm × 0.83 mm in-plane spatial resolution, through-plane resolution of 1.6 mm and a flip angle of 10 degrees. The excitation volume in the superior/inferior direction was customized for each subject to include all their breast tissue. Separate fat and water images were reconstructed from these sequences and further processing was performed to remove noise while preserving edge information using an adaptive template filter that optimizes the filter shape and coefficients on a voxel-by-voxel basis as described by Ahn et al. [72]. To simulate proton density weighting, these images were scaled to produce approximately equal signal levels in the fat and fibroglandular tissues. These images served as the base anatomical reference for the Anatomy Module. The Anatomy Module was then set up as follows: the fat tissue was assigned a nine-peak spectral model corresponding to human subcutaneous fat [73] and a T1 value of 296 ms, as described in the literature, for breast adipose tissue at 1.5T [74]. The water tissue was assumed to be completely on-resonance. The water tissue was divided into skin, muscle and fibroglandular tissue subtypes, using a previously described breast segmentation algorithm [75], and assigned T_1_ values of 887 ms [76], 1130 ms [77], and 1266 ms [74], respectively, based on values reported in the literature. Simulated regions of BPE were added to the fibroglandular tissue representing minimal, mild, moderate, and marked levels of enhancement.

#### 2.6.2. Simulation 2

Simulations were performed using the functionality of the Physiology Module described above. Four lesions, one for each morphologic type, were inserted into the simulated breast tissue. Lesion enhancement characteristics were defined by the three parameter Generalized Kinetic Model (GKM) [8], which is defined as:

(4) $$C_{t}(t)=K^{trans}\left(C_{p}(t)⊗\exp\left(-k_{ep}(t)\right)\right)+v_{p}C_{p}(t)$$

where *C_t_*(*t*)is the tissue contrast agent concentration, *C_p_*(*t*) is the vascular input function, *K^trans^* is the volume transfer constant between the blood plasma and the extravascular extracellular space (EES), *k_ep_* = *K^trans^*/*v_e_* is the transfer rate constant between the EES and the blood plasma, *v_e_* is the fractional EES and *v_p_* is the fractional plasma volume. The vascular input function *C_p_*(*t*) was simulated using the publicly available dispersion model described by Barboriak et al. [78]. Lesion parameters were assigned as follows: diameters of 1 cm, 1.25 cm, 1.25 cm and 1.5 cm for the round, lobulated, irregular and spiculated lesions, respectively, and all lesions were assigned to have homogeneous spatial enhancement following the curve, specified by *K^trans^* = 0.08 min^−1^, *v_e_* = 0.4, and *v_p_* = 0. 

#### 2.6.3. Simulation 3

The MRI System Module was set up to simulate two different types of fat suppression. The simulation was initially set up as described for Simulation 1 above. The simulation was first performed using a chemically selective fat saturation pulse with a flip angle of 100 degrees and a TR of 500 ms. The simulation was performed a second time with no fat saturation applied. Data were collected with an echo time of 2.2 ms and 4.2 ms, corresponding to the opposed-phase and in-phase echo times for imaging at 1.5T. These echo times are typically used for reconstructions making use of a two-echo CSE-MRI approach to generate fat and water separated images. 

#### 2.6.4. Simulation 4

The Simulator Module was used to simulate varying levels of complex noise. The simulation was initially set up as described for Simulation 1. Three sets of output data were collected, one with no simulated noise, one with white Gaussian noise added to produce an SNR of 30, and one with white Gaussian noise added to produce an SNR of 15. SNR settings were selected to allow for a visual appreciation of the different noise levels in the resulting reconstructed images.

#### 2.6.5. Simulation 5

In the final simulation, a single simulated spiculated lesion with a diameter of 2 cm in the longest direction was inserted into the fibroglandular tissue. The lesion was assigned to display rim enhancement with the edges of the lesion following a washout enhancement curve defined by *K^trans^* = 0.5 min^−1^, *v_e_* = 0.3, and *v_p_* = 0 while the interior of the lesion displayed slower uptake and persistent enhancement, as defined by the curve *K^trans^* = 0.08 min^−1^, *v_e_* = 0.4, and *v_p_* = 0. A rectilinear Cartesian sampling pattern consisting of a 256 × 256 × 100 encoding matrix and a TR of 7.8 ms acquired in 3.3 min per time frame was simulated to represent a conventional MRI acquisition without acceleration. This simulation was repeated with 3.3 min time frames but all the simulated k-space data were produced using the temporal information at the center of the time frame, which is what the data would look like if all k-space information could be acquired instantaneously. This represents the true or gold standard enhancement behavior for the simulation. An additional pair of simulations were performed where it was assumed the same sampling pattern could be acquired in 30 s. 

## 3. Results

The four modules of the breast DRO allow a customized simulation of a breast DCE simulation to be set up and run. The results of the simulations below demonstrate the functionality of each of the modules.

### 3.1. Simulation 1

**Figure 3:**
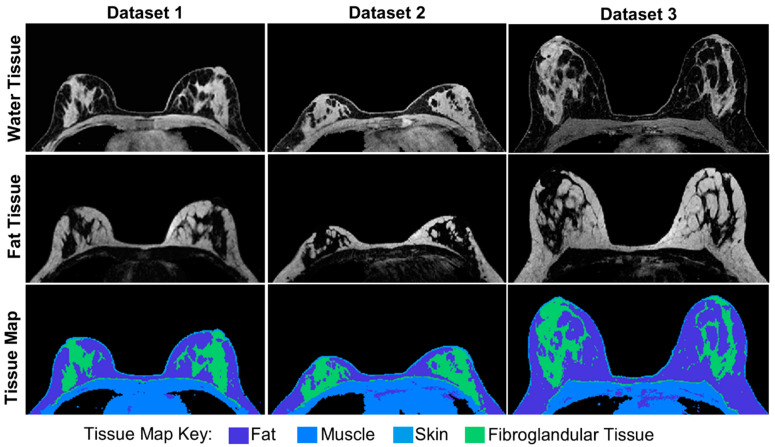
Base anatomical images for three different datasets. Images used to generate the water tissue are shown in the top row while images used to generate the fat tissue are shown in the middle row. The water tissue was divided into three tissue subtypes: muscle, skin, and fibroglandular tissue, as shown in the tissue map in the bottom row. Fat is a distinct tissue from the water tissue but is included in the tissue map for clarity.

Figure 3 shows the output of the Anatomy Module using base anatomical images from three volunteers. In this implementation, the fat tissue was treated as one uniform tissue throughout the volume of interest. The water tissue was divided into skin, muscle, and fibroglandular tissue subtypes, as shown in Figure 3. The masks used to define the extent of the different tissues are continuous, allowing a voxel to be represented as a mixture of fat and water tissue. Water tissue subtypes are represented by a combination of binary and continuous masks, allowing for some features to be a weighted combination of different tissue subtypes and others to be strictly one tissue type.

**Figure 4:**
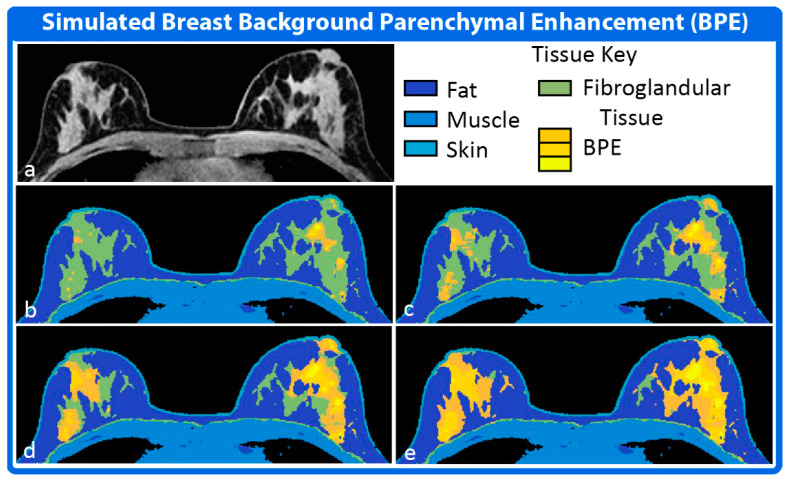
Simulated Breast Background Parenchymal Enhancement (BPE). The image space representation of the water tissue is shown in (**a**). Regions corresponding to specific tissue sub-types of the water tissue are displayed in the corresponding colormaps (**b**–**e**). Fat is a distinct tissue from the water tissue but is shown in the color maps for clarity. BPE is a tissue sub-type of the fibroglandular tissue and was preferentially assigned to the regions of fibroglandular tissue in the upper outer quadrants of the breast. Simulated regions of BPE representing minimal (**b**), mild (**c**), moderate (**d**) and marked (**e**) BPE are shown.

Figure 4 shows the results of applying simulated BPE to dataset 1 from Figure 3. The BPE is represented as multiple, concentric water tissue subtypes all contained within the region occupied by the fibroglandular tissue. Each BPE subtype is represented by its own unique modeling parameters, producing different enhancement profiles in the Simulator Module. 

### 3.2. Simulation 2

**Figure 5:**
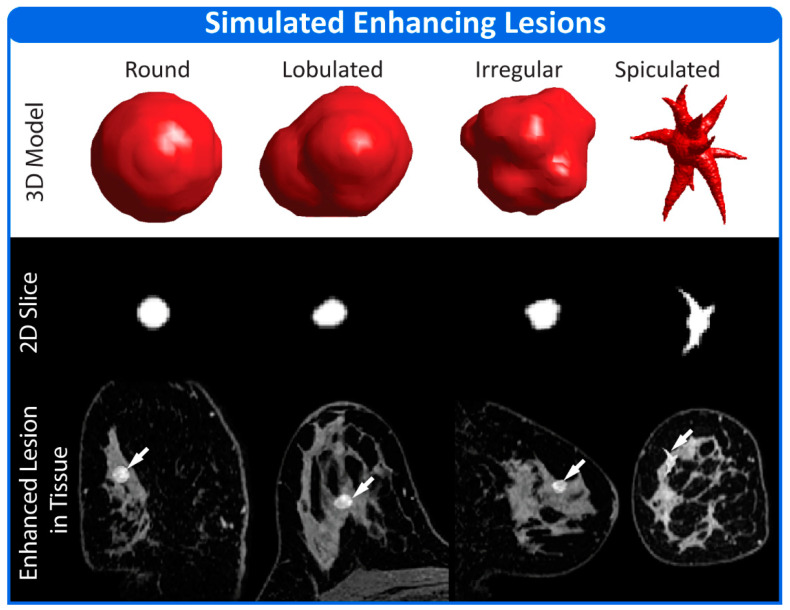
Four lesions showing the representative shapes produced by the breast DRO phantom are depicted. A 3D model of the lesion morphology is shown in the top row. The middle row shows a 2D cross section of the lesion taken through the center of the lesion. The bottom row shows the same central slice of the lesion as it appears inserted into the base anatomical images contained in the Anatomy Module. Lesions are shown as enhanced to make them more visually apparent.

The four simulated, enhancing lesions are shown in Figure 5. Lesions were preferentially placed in the fibroglandular tissue but allowed to extend into the fat tissue, as can be seen with the spiculated lesion.

### 3.3. Simulation 3

**Figure 6:**
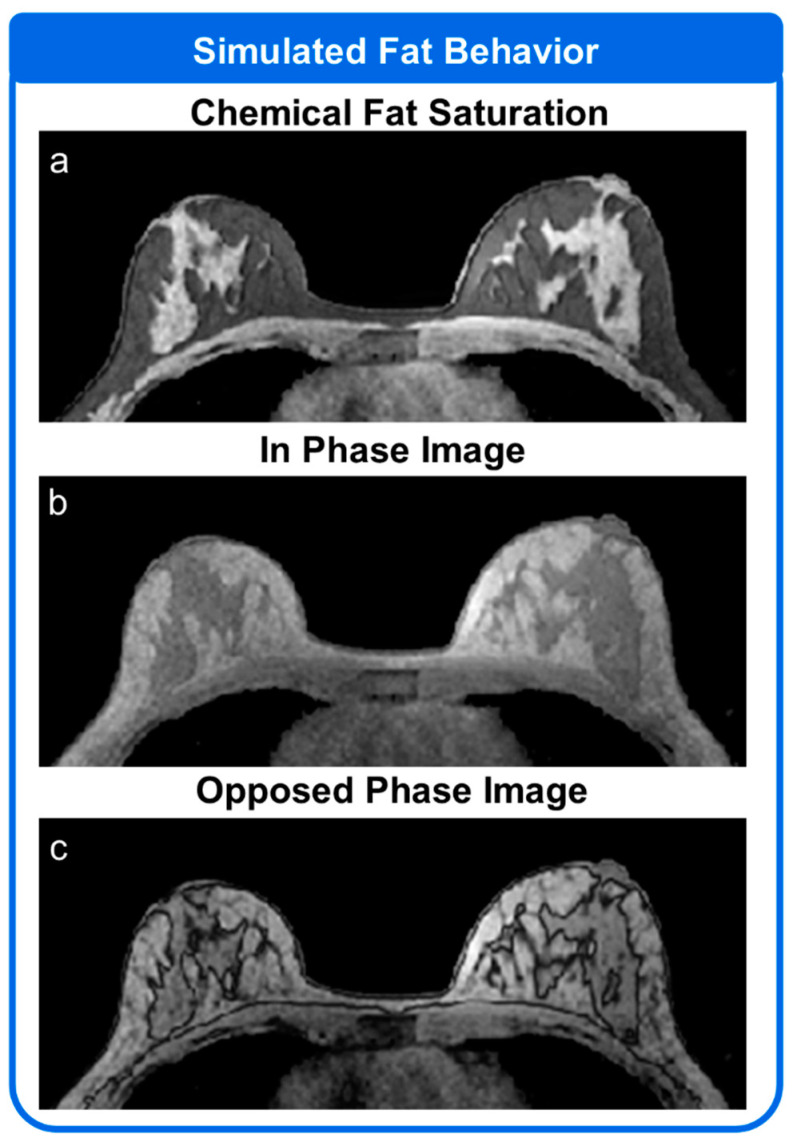
Simulation results utilizing different approaches for fat saturation. (**a**) The results of chemical fat saturation using a periodic inversion pulse is simulated. Simulated (**b**) in-phase and (**c**) opposed-phase images appropriate for use in two-echo CSE approaches using the same dataset are also shown.

Figure 6 shows the results of simulations performed using two different approaches to removing the fat signal. Dataset 1 from Figure 3 was used as the base dataset for the simulation. In Figure 6a the simulated fat signal is largely suppressed with low levels of fat signal leaking through in the fat regions. Figure 6b,c show the simulated fat tissue at different echo times. In Figure 6b, the fat and water are in phase. In Figure 6c, the fat and water have opposing phases resulting in the characteristic India-ink artifact at the tissue interfaces.

### 3.4. Simulation 4

**Figure 7:**
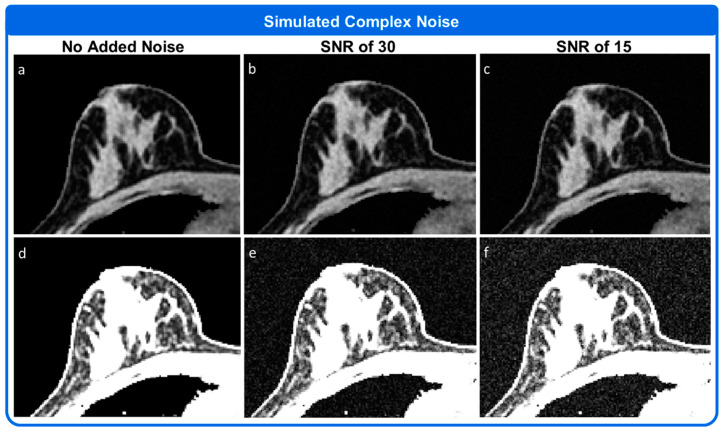
Addition of simulated complex Gaussian noise. The top row shows the simulated breast images with (**a**) no additional simulated noise, (**b**) simulated complex white Gaussian noise added to produce and SNR of 30, and (**c**) simulated white Gaussian noise added to produce an SNR of 15. The same images are shown below (**d**–**f**) with image intensity set to allow visualization of the noise in the background. All images within a single row are displayed using the same color scale.

Figure 7 shows the results of adding complex, white gaussian noise to dataset 1 from Figure 3. Complex noise is added to the k-space data prior to the k-space data being returned to the user. Once reconstructed, the simulated white Gaussian noise is seen both in the simulated breast tissue and in the background, as shown in Figure 7. 

### 3.5. Simulation 5

**Figure 8:**
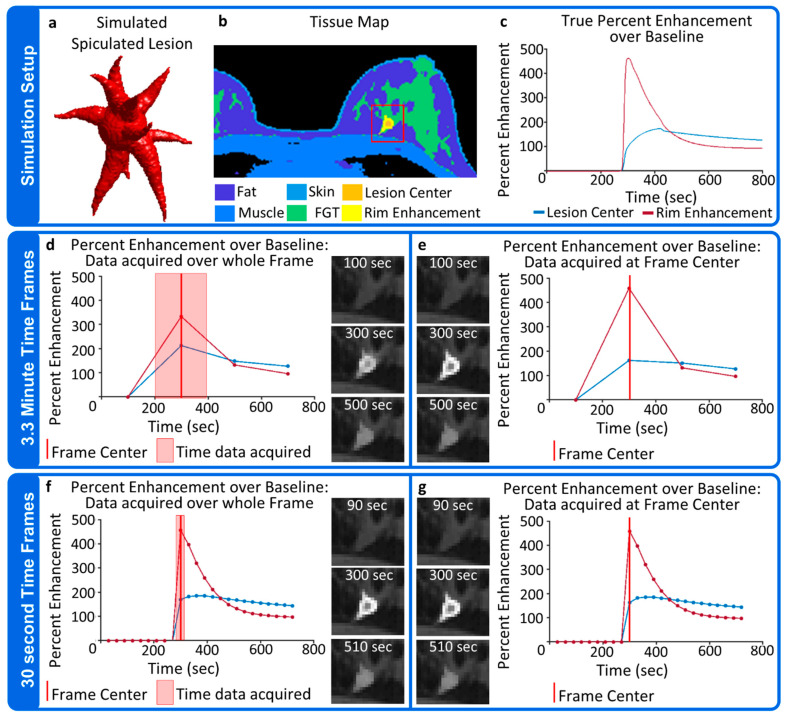
Simulation set up (top row) and results produced for running a simulation consisting of (**a**) a simulated enhancing spiculated lesion (**b**) inserted into the fibroglandular tissue and (**c**) displaying a persistently enhancing center region surrounded by rim enhancement with a characteristic washout curve. The simulation was run with 3.3 min time frames where a single frame is made up of k-space lines acquired at different points over the course of the time frame, as represented by the red overlay on the plot in (**d**). Enlarged images of the region contained in the red box in (**c**) are shown below the plot for each time point. A corresponding truth dataset (**e**) consisting of k-space data generated temporal data from the center of the time frame only is generated for comparison. The simulation was repeated (**f**) with 30 second time frames and (**g**) corresponding truth dataset generated at the center of each time frame.

The set-up for Simulation 5 is illustrated in Figure 8a–c. A simulated spiculated lesion displaying rim-enhancement with a washout enhancement curve surrounding a central region with a slower uptake and more persistent enhancement over the course of the simulation was placed in the fibroglandular tissue. Figure 8d,e show the results when sampling the simulated data every 3.3 min, a frame rate that could be achieved with conventional encoding techniques without including acceleration techniques of any kind. The first post-contrast time frame was centered such that the enhancing rim reached its max value as the center of the k-space was being sampled while the higher spatial frequencies were acquired when the lesion showed lower levels of enhancement. As seen in Figure 8d, this averaging of temporal information across the time frame leads to an underestimation of the rim enhancement as compared to the true level of enhancement shown in Figure 8e. The effects of temporal averaging are also seen in the center of the lesion, with the lesion core showing greater enhancement in Figure 8d than is seen in Figure 8e, which shows the true level of enhancement. Figure 8f,g show the results when the simulation was performed with 30 s time frames. In reality, full resolution frame rates on the order of 30 s cannot be achieved without the use of accelerated imaging techniques, but they can still be explored using the DRO. In the 30 s per frame simulation, the impact of temporal averaging is greatly reduced due to the shorter acquisition time. As expected, much more accurate curve shapes can be reproduced with the shorter time frames. 

## 4. Discussion

This work described a framework for generating a numerical breast DRO for use in analyzing and optimizing DCE breast MRI data acquisition and reconstruction techniques. The DRO consists of four modules which control different aspects of the simulation including the appearance and physiological behavior of the breast tissue, as well as the MRI system settings and parameters. These modules are combined into a pipeline to produce simulated k-space data of a DCE breast exam. The DRO uses images from in vivo breast MRI exams to form a base anatomical model for the simulation. Each tissue in the breast DRO is assigned a unique set of parameterized model inputs to allow for the simulation of different physiological responses. In this work, fat and water images were used as the base anatomical model and the overall fat fraction was calculated on a voxel-by-voxel basis. Fat was modeled using a 9-peak model for subcutaneous fat and tissue parameters were defined using literature values for T_1_ relaxation and modeling parameters consistent with the GKM. However, other parameters are possible or entirely different models may be chosen to represent these behaviors. Features of interest such as breast BPE and simulated lesions representing common morphological shapes have been incorporated into the DRO independently of the base anatomical model, allowing the simulation of these features with their own model specific parameters. The spatial distribution of the modeling parameters assigned to the simulated lesions can produce homogeneous, heterogenous or rim enhancement behavior. The simulated DCE acquisition is controlled through user defined selections for MRI parameters including TR, flip angle, field strength, acquisition trajectory and acquisition timing information. The final output of the DRO is simulated k-space data, which can be reconstructed via a method of the user’s choice. Unlike in vivo testing, the underlying enhancement characteristics of the breast DRO are known, allowing for analysis of the errors introduced by the chosen acquisition or reconstruction method. 

Researchers within the quantitative imaging community, including the RSNA associated QIBA and the ISMRM associated OSIPI groups, have described the need for the development and use of validation tools, including numerical simulations, to characterize the repeatability and reproducibility of quantitative imaging biomarkers [1,2,3]. The breast DRO described in this work has already been used to validate a few specific novel acquisition and reconstruction strategies [31,55,56,57,58,59,60], however, it can be readily used for alternative novel DCE approaches. The use of numerical simulations is particularly important in settings such as breast imaging where relatively long contrast agent wash-out times require many hours to a day between consecutive injections, preventing evaluation of agreement between different acquisition strategies. Recent awareness of gadolinium deposition has raised additional concerns around contrast agent usage during the development of novel imaging methods [79]. These challenges are even more relevant in the setting of multi-center trials when attempting to characterize differences between vendor specific DCE implementations where relevant spatial-temporal sparsity must be controlled for.

In creating a DRO such as the digital breast phantom, compromises must be made between realism and computational complexity. The DRO includes several important features that are seen in vivo, including intra-voxel mixtures of fat and fibroglandular tissue, normal enhancing breast BPE, and enhancing lesions. While all these features provide a more complex simulation environment for testing and validation purposes, they do not represent the true complexity seen in vivo. The spatial distribution of the fat and fibroglandular tissue is determined from breast MRI images, and is thus limited by the resolution of original acquisition. Higher resolution images would provide greater complexity but would also increase memory usage and computation time. The included simulated lesions are relatively simple and stylized. They are not intended to exactly mimic those seen in vivo, as might be desired in applications investigating a human reader’s ability to detect lesions. Instead, they provide dynamic high and low spatial frequency information to allow for analysis of the ability of an accelerated imaging method to capture both the spatial and temporal features of interest. Realistic enhancing lesions have been produced in DRO’s through the incorporation of in vivo imaging data from enhancing lesions, as described by Bosca et al. [36]. If more realistic enhancing lesions are required, the same process may be applied to the DRO presented in this work. Additional compromises were made between the complexity of the representation of spatial and temporal features of the breast imaging environment and the computational complexity of the simulation pipeline. All dynamic processing is performed in k-space on a tissue-by-tissue basis as described in the methods section. This allows for the efficient simulation of both rapid temporal processes, such as fat signal precession following the excitation pulse, and long duration processes, such as simulated enhancement. This approach also limited the number of Fourier transforms required, which increased the speed of the processing pipeline. However, this limits the ability of this DRO to efficiently simulate spatially varying effects such as B0 and B1 inhomogeneities and non-ridged patient motion. Such effects are not currently included in the DRO. Despite these compromises, the framework presented here still provides a reasonably realistic, complex enhancing environment allowing for the visualization and analysis of the impacts of different sampling and reconstruction strategies, which would be challenging or impossible to directly compare in vivo. Insights gained from analyses such as these may help guide further in vivo validation efforts of accelerated MRI techniques in preclinical or clinical models. 

One of the strengths of the DRO framework is the flexible and modular design. The same base anatomical image set can be used to simulate a variety of imaging conditions. The current implementation only includes base breast images from three volunteers. However, future efforts will aim to add additional data from different body habitus as well as coil sensitivity maps from different coils geometries. A comparison of different imaging methodologies in the presence of motion is often hampered by the need for reproducible movement patterns, but numerical simulations have been used to characterize novel imaging methods in body imaging applications [42,45,80]. Motion studies have been performed in the setting of conventional prone imaging [81] as well as supine imaging [82], and we aim to include motion capabilities into the breast DRO in the future. Although this manuscript focuses on the clinical application of breast imaging, the modules for the base images and contrast enhancement patterns are extensible to other clinical settings [60].

## 5. Conclusions

We have described the design of a breast DRO that serves as a framework for analyzing, optimizing, and comparing accelerated DCE breast imaging acquisition and reconstruction algorithms. While the current work is focused on breast MRI, the same process could be used to create DRO’s for DCE in other areas of the body. The use of such DROs could aid in the optimization and validation process of accelerated DCE MRI sequences and help guide in vivo validation efforts. 

## Data Availability

The code presented here is publicly available at https://github.com/lchenze/DRO_Breast_DCE_MRI (accessed on 24 March 2022).
